# A retrospective study of autologous hematopoietic stem cell transplantation for peripheral T-cell lymphoma: pre-transplant patients with partial remission benefit from post-transplant maintenance therapy

**DOI:** 10.3389/fonc.2023.1162413

**Published:** 2023-05-15

**Authors:** Zhenghua Huang, Zhen Li, Juan Wang, Ruirui Gui, Yingling Zu, Fengkuan Yu, Quande Lin, Huifang Zhao, Yanli Zhang, Baijun Fang, Yanyan Liu, Keshu Zhou, Yufu Li, Yuewen Fu, Zhihua Yao, Yongping Song, Jian Zhou

**Affiliations:** ^1^ Department of Hematology, Affiliated Cancer Hospital of Zhengzhou University and Henan Cancer Hospital, Zhengzhou, China; ^2^ Department of Hematology, The First Affiliated Hospital of Zhengzhou University, Zhengzhou, China

**Keywords:** peripheral T-cell lymphoma, autologous stem cell transplantation, maintenance therapy, efficacy, partial remission

## Abstract

**Background:**

Whether autologous hematopoietic stem cell transplantation (ASCT) improves the survival of patients with peripheral T-cell lymphoma (PTCL) remains controversial. Some studies have demonstrated that the efficacy of ASCT is superior in patients with complete remission (CR), whereas patients with partial remission (PR) remain vulnerable to relapse after ASCT, resulting in decreased survival rates. Maintenance therapy after chemotherapy may reduce the relapse rate of PTCL and improve survival; however, the role of maintenance therapy after ASCT in PTCL remains unclear. In this study, we aimed to analyze the efficacy of ASCT and post-transplant maintenance therapy in PTCL.

**Methods:**

We retrospectively analyzed the clinical data of 69 patients with PTCL who underwent ASCT at our center between November 2001 and November 2021. According to the patients’ intention, thirty patients received post-transplant maintenance treatment, whereas 39 did not. The overall survival (OS) and progression-free survival (PFS) between the groups were compared using the log-rank test.

**Results:**

At a median follow-up of 36 months, the entire cohort’s 3-year OS and PFS were 67.8% and 53.0%, respectively. The 3-year OS and PFS of patients with CR1, CR2, and PR were 85.3% and 65.4%, 80.0% and 60.0%, and 38.4% and 32.0%, respectively (OS: *P*=0.001; PFS: *P*=0.003). The relapse rates between the groups with or without maintenance therapy were 26.7% vs. 52.2%, the 3-year OS was 86.0% vs. 54.2% (*P*=0.004), and the 3-year PFS was 73.3% vs. 37.5% (*P*=0.004). Further analysis revealed that the efficacy of maintenance therapy was not significant in patients with CR1 and CR2, whereas patients with PR benefited from maintenance therapy. The relapse rate of patients with PR who received or did not receive maintenance therapy was 33.3% vs. 78.7%, 3-year OS was 66.7% vs. 21.9% (*P*=0.007), and 3-year PFS was 66.7% vs. 12.5% (*P*=0.004).

**Conclusions:**

Patients with CR in PTCL benefit from ASCT, and post-transplant maintenance therapy reduces the relapse rate and significantly improves OS and PFS in patients with PR.

## Introduction

Peripheral T-cell lymphoma (PTCL) is a type of malignant proliferative disease that originates from mature T lymphocytes and is characterized by high heterogeneity and aggressiveness. It accounts for approximately 10% of non-Hodgkin’s lymphoma, with a higher proportion (20–25%) in Asia than in Western countries. Common PTCL subtypes include natural killer/T-cell lymphoma (NKTCL), angioimmunoblastic T-cell lymphoma (AITL), anaplastic large-cell lymphoma (ALCL), and peripheral T-cell lymphoma-not otherwise specified (PTCL-NOS). Currently, anthracycline-based CHOP (vincristine, adriamycin, cyclophosphamide, and prednisone) or CHOP-like (vincristine, adriamycin, cyclophosphamide, prednisone, and etoposide) chemotherapy regimens are still used as first-line treatment, and the overall response rate can reach 70–80%. However, the relapse rates of PTCL are high, and the long-term survival rates are low, with 5-year overall survival (OS) rates of only 35–40% (1). Autologous hematopoietic stem cell transplantation (ASCT) has been increasingly studied to improve survival rates. Both the National Comprehensive Cancer Network and the European Group for Blood and Marrow Transplantation recommend ASCT as the first-line consolidation therapy for some aggressive lymphomas and salvage therapy for relapsed or refractory lymphomas. Owing to the low prevalence and high heterogeneity of PTCL, the efficacy of ASCT for PTCL remains controversial (2, 3), and the role of post-transplant maintenance therapy remains unclear. A previous study revealed that maintenance treatment with chidamide after chemotherapy reduced relapse and improved survival in patients with PTCL (4), suggesting that post-transplant maintenance therapy with histone deacetylation inhibitors (HDAC) such as chidamide can further reduce the relapse rate and improve survival. Here, we conducted a single-center retrospective study to analyze the outcomes of patients with PTCL who received ASCT and to further explore the role of post-transplant maintenance therapy in PTCL.

## Materials and methods

### Patients

In this single-center retrospective study, we included 436 patients with PTCL who were treated at our center between November 2001 and November 2021. All patients were diagnosed with PTCL based on pathological cytomorphology and immunohistochemistry (pathological subtypes were classified according to “the 2008 WHO classification of tumors of hematopoietic and lymphoid tissues”, and pre-2008 cases were reclassified). After excluding 107 patients with ALK + ALCL subtype or the subtypes of less than five cases, 81 refractory patients, 109 patients not eligible for transplantation (age > 65 years, Eastern Cooperative Oncology Group (ECOG) score > 2, organ dysfunction, or active infection), 59 patients who refused transplantation, and 11 patients who received allogeneic hematopoietic stem cell transplantation, 69 patients with PTCL who received ASCT were finally enrolled **Supplementary Material**. Pro-transplant maintenance therapy was recommended for 56 patients who met at least one of the following criteria: stages III-IV, International Prognostic Index score (IPI) > 2, and failure to achieve complete remission (CR) after transplantation. According to the patients’ intention, 30 of 56 patients eventually received maintenance treatment, including 19 patients with pre-transplant CR1, 2 with CR2, and 9 with partial remission (PR). Depending on the patients’ intention, remission status was assessed by positron emission tomography-computed tomography or computed tomography, and bone marrow puncture was additionally performed on patients who had bone marrow invasion previously. The efficacy evaluation was based on the 2014 version of the Lugano Efficacy Evaluation Criteria for non-Hodgkin’s lymphoma. This study was approved by the Ethics Committee of the Henan Cancer Hospital, and all patients provided written informed consent.

### Treatment procedure

Stem cells were mobilized and collected using an induction chemotherapy regimen combined with granulocyte colony-stimulating factor (G-CSF) within 3 months before transplantation. All 69 patients underwent complete conditioning regimens; 13 patients were treated with a regimen containing total body irradiation, 41 were treated with BEAM (carmustine, etoposide, cytarabine, and melphalan), 9 were treated with BEAC (carmustine, etoposide, cytarabine, and cyclophosphamide), and 6 were treated with CEAM (semustine, etoposide, cytarabine, and melphalan).

Post-transplant maintenance therapy for patients with platelet count > 30 × 10^9^/L and neutrophil count > 1.0 × 10^9^/L was started 3 months after transplantation and lasted until the patients relapsed, progressed, or reached 2 years. Two maintenance regimens were used in this study; 20 mg chidamide orally twice a week (at intervals > 3 days) and 100–150 mg thalidomide orally at bedtime per day. Patients who were administered chidamide underwent routine blood tests weekly and suspended the drug when grades 3–4 myelosuppression (platelets < 30×10^9^/L or neutrophils < 1.0×10^9^/L) occurred. Maintenance treatment continued after grades 3–4 myelosuppression resolved with G-CSF, thrombopoietin, or other supportive therapies. Patients who did not develop grades 3–4 myelosuppression within 1 month underwent routine blood tests monthly. Patients administered thalidomide were closely monitored for adverse events (AEs) related to the gastrointestinal tract and peripheral nervous system, and clinical intervention was provided as required. All patients were evaluated for disease status every 3 months after transplantation.

### Statistical analysis

The primary endpoints observed in this study were relapse and progression rates, OS, and progression-free survival (PFS). Distribution differences in clinical characteristics between the groups were analyzed using the chi-square test. The probabilities of OS and PFS were estimated using the Kaplan–Meier method, and differences between the groups were compared using the log-rank test. Multivariate analysis was performed using the Cox proportional hazards model. All statistical tests were two-sided, and statistical significance was considered at *P <*0.05. Statistical analyses were performed using SPSS version 26.0 (SPSS Inc).

## Results

### Characteristics of the study participants

Forty-six males (66.7%) and 23 females (33.3%) with PTCL were included in this study, with a median age of 40 (12–62) years. The histological subtypes included NKTCL (n=28, 40.6%), AITL (n=13, 18.8%), ALCL (ALK-) (n=7, 10.2%), and PTCL-NOS (n=21, 30.4%). Pre-transplant disease remission reached CR in 44 cases (39 cases with CR1 and 5 cases with CR2) and PR in 25 cases. All patients were scored using the IPI and Prognostic Index for T-cell lymphoma scores. No significant differences were observed in the clinical characteristics between the maintenance and non-maintenance groups (**Table 1**).

**Table 1:** Characteristics of 69 Patients With PTCL .

Characteristics	Number of Patients N(%)	Number of patients in maintenance treatment N(%)	Number of patients in no maintenance treatment N(%)	*P*
Gender				0.606
Male	46(66.7)	19(63.3)	27(69.2)	
Female	23(33.3)	11(36.7)	12(30.8)	
Age (Years)				1.000
Median (range)	40(12-62)	46 (19–62)	31 (12–60)	
>60 years	2(2.9)	1(3.3)	1(2.6)	
B symptoms				0.966
Yes	32(46.4)	14(46.7)	18(46.2)	
No	37(53.6)	16(53.3)	21(53.8)	
Extranodal involvement				0.384
Yes	54(78.3)	22(73.3)	32(82.1)	
No	15(21.7)	8(26.7)	7(17.9)	
LDH				0.345
Elevated ( >240U/L)	25(36.2)	9(30.0)	16(41.0)	
Normal (≤240U/L)	44(63.8)	21(70.0)	23(59.0)	
ALC				0.166
Elevated	6(8.7)	1(3.3)	5(12.8)	
Normal	63(91.3)	29(96.7)	34(87.2)	
β2-MG				0.815
Elevated ( >3.0mg/L)	38(55.1)	17(56.7)	21(53.8)	
Normal (≤3.0mg/L)	31(44.9)	13(43.3)	18(46.2)	
Ann-Arbor stage				0.305
II	13(18.8)	4(13.3)	9(23.1)	
III-IV	56(81.2)	26(86.7)	30(76.9)	
Bone marrow involvement				0.401
Yes	7(10.1)	2(6.7)	5(12.8)	
No	62(89.9)	28(93.3)	34(87.2)	
Pathological subtype				0.054
NKTCL	28(40.6)	9(30.0)	19(48.7)	
AITL	13(18.8)	10(33.3)	3(7.7)	
ALCL ALK-	7(10.2)	3(10.0)	4(10.3)	
PTCL-NOS	21(30.4)	8(26.7)	13(33.3)	
Status before ASCT				0.598
CR1	39(56.5)	19(63.3)	20(51.3)	
CR2	5(7.2)	2(6.7)	3(7.7)	
PR	25(36.2)	9(30.0)	16(41.0)	
SD/PD	0	0	0	
IPI score				0.945
0-2	48(69.6)	21(70.0)	27(69.2)	
3-5	21(30.4)	9(30.0)	12(30.8)	
PIT score				0.600
≥3	63(91.3)	28(93.3)	35(89.7)	
<3	6(8.7)	2(6.7)	4(10.3)	

PTCL, Peripheral T-cell lymphoma; ASCT, autologous hematopoietic stem cell transplantation; B symptoms, fever, night sweats, weight loss; LDH, lactate dehydrogenase; ALC, absolute lymphocyte count; β2-MG, β2-microglobulin; NKTCL, natural killer/T-cell lymphoma; AITL, angioimmunoblastic T-cell lymphoma; ALCL, anaplastic large-cell lymphoma; ALK-, anaplastic lymphoma kinase negative; PTCL-NOS, Peripheral T-cell lymphoma, not otherwise specified; CR1, first complete remission; CR2, second complete remission; PR, partial remission; SD, stable disease; PD, progressive disease; IPI score, International Prognostic Index; PIT, Prognostic Index for T-cell lymphoma.

### Treatment before transplantation

Among the 69 included patients, 44 achieved CR1 after first-line chemotherapy (39 patients remained in CR1, the other 5 patients relapsed within 2 years and achieved CR2 after second-line chemotherapy), 20 achieved PR after second-line chemotherapy, and 5 achieved PR after third-line chemotherapy. The first-line chemotherapy regimens included CHOP, CHOP-like, and DICE (cisplatin, ifosfamide, etoposide, and dexamethasone with or without L-asparaginase/pegaspargase). The second- and third-line chemotherapy regimens included DICE, Hyper-CVAD, cyclophosphamide, vincristine, doxorubicin, and dexamethasone alternating with methotrexate and cytarabine, GDP (gemcitabine, dexamethasone, and cisplatin), DHAP (dexamethasone, cytarabine, and cisplatin), ESHAP (etoposide, methylprednisolone, cytarabine, and cisplatin), GEMOX-L (gemcitabine, oxaliplatin, and L-asparaginase/pegaspargase), and SMILE (dexamethasone, methotrexate, ifosfamide, etoposide, and L-asparaginase/pegaspargase).

### Engraftment and toxicity

Infusion doses of mononuclear cells and CD34+ cells were (7.36 ± 0.58) ×10^8^/kg and (8.53 ± 1.21) ×10^6^/kg, respectively. Sixty-six patients achieved sustained myeloid engraftment, except for 3 in whom platelet engraftment failed. The median days to neutrophil and platelet engraftment were 10 (range: 8–61) and 13 (range: 6–37), respectively. The transplantation-related AEs included fever (84.5%), nausea and vomiting (74.1%), diarrhea (72.4%), oral ulcers (58.6%), and hepatotoxicity (6.8%).

### Response and survival

**Figure 1:**
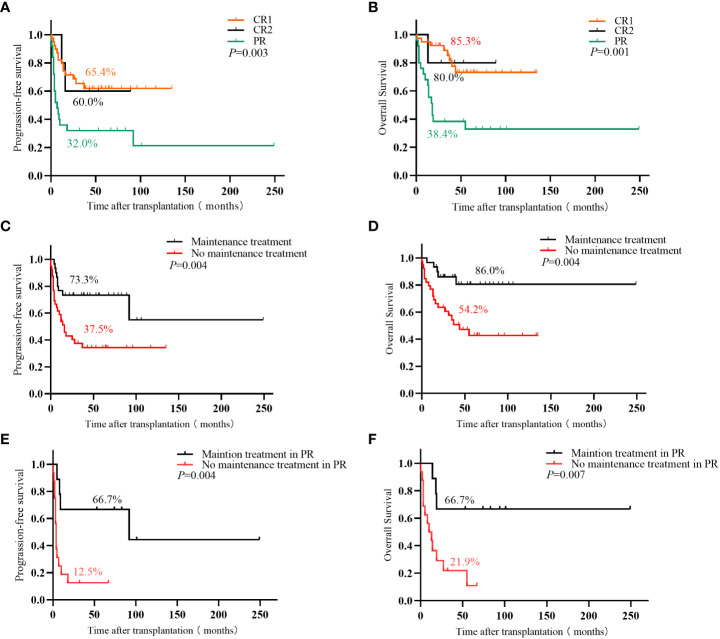
**(A)** Progression-free survival and **(B)** overall survival in PTCL patients with different disease states before ASCT; **(C)** Progression-free survival and **(D)** overall survival of PTCL patients with or without maintenance treatment in the entire cohort; **(E)** Progression-free survival and **(F)** overall survival in PR patient with or without maintenance treatment.

Sixty-one patients achieved CR after transplantation, 18 of whom had PR before transplantation. The CR rates before and after transplantation were 56.5% and 88.4%, respectively. With a median follow-up of 36 months (1–175 months), 44 patients survived, and 25 died. Among 27 patients with relapse or progression, 5 achieved CR after chemoradiotherapy, 1 achieved CR after allogeneic hematopoietic stem cell transplantation, 3 achieved PR after treatment with chidamide or thalidomide, and 18 died. Of the remaining 7 patients who died, 6 died of infection, and 1 died of platelet engraftment failure. The 3-year OS and PFS were 67.8% and 53.0%, respectively. Furthermore, the survival of patients with CR1 was significantly higher than that of patients with PR (3-year OS: 85.3% vs. 38.4%, *P*<0.001; 3-year PFS: 65.4% vs. 32.0%, *P*=0.001). Patients with CR2 had a survival trend better than patients with PR (3-year OS: 80.0% vs. 38.4%, *P*=0.123; 3-year PFS: 60.0% vs. 32.0%, *P*=0.158) (**Figure 1**).

### Maintenance therapy after transplantation

Thirty patients (19 with pre-transplant CR1, 2 with CR2, and 9 with PR) received maintenance therapy (20 were treated with chidamide, and 10 with thalidomide), and 9 cases of relapse and progression. Among these 30 patients, 19 completed the maintenance therapy course with 1 patient (3.3%) relapsing, 3 patients were on maintenance therapy with none relapsing, and 8 patients (26.7%) stopped maintenance therapy due to relapse and progression. The median time of relapse and progression was 8 (4–14) months. Thirty-nine patients did not receive maintenance treatment, and relapse and progression were observed in 18 patients, of which 15 (38.5%) relapsed and progressed within 2 years of transplantation, and the median time to relapse and progression was 7 (1–18) months. The remaining 3 patients (7.7%) relapsed beyond 2 years after transplantation. The 3-year relapse rates between the groups with and without maintenance therapy were 26.7% vs. 52.2%, the 3-year OS was 86.0% vs. 54.2% (*P*=0.004), and the 3-year PFS was 73.3% vs. 37.5% (*P*=0.004), respectively. Patients with PR benefited significantly from maintenance treatment than those with CR1 and CR2. The 3-year relapse rate of patients with PR who received or did not receive maintenance was 33.3% vs. 78.7%, the 3-year OS was 66.7% vs. 21.9% (*P*=0.007), and the 3-year PFS was 66.7% vs. 12.5% (*P*=0.004), respectively (**Table 2**; **Figure 1**).

**Table 2:** Maintenance treatment after transplantation.

Disease status before transplantation	Relapse rate		3-year OS			3-year PFS		
	Maintenance Treatment^1^ (%)	No treatment^2^ (%)	Maintenance Treatment (%)	No treatment (%)	*P*	Maintenance Treatment (%)	No treatment (%)	*P*
CR1	26.3	34.0	94.7	78.1	0.281	73.7	58.2	0.326
CR2	0	66.7	100.0	66.7	0.414	100.0	33.3	0.199
PR	33.3	78.7	66.7	21.9	**0.007**	66.7	12.5	**0.004**
Total	26.7	52.2	86.0	54.2	**0.004**	73.3	37.5	**0.004**

OS, overall survival; PFS, Progression-free survival; CR1, first complete remission; CR2, second complete remission; PR, partial remission;1: with maintenance treatment;2: no maintenance treatment. bold font, *P*<0.05.

### Maintenance treatment AEs

All patients endured maintenance treatment, and none discontinued treatment due to serious AEs. Among the 20 patients who received maintenance treatment with chidamide, neutropenia and thrombocytopenia occurred in 9 (45.0%) and 12 (60.0%) patients, respectively. Two patients (10.0%) and 5 patients (25.0%) suspended maintenance treatment due to decreased neutrophil (< 1.0×10^9^/L) and platelet (< 30×10^9^/L) counts, respectively. All patients recovered after G-CSF and TPO administration, and continued maintenance treatment. Eight patients (40.0%) experienced fatigue, and 7 (35.0%) experienced gastrointestinal reactions, such as nausea, vomiting, and diarrhea. The 10 patients that received maintenance therapy with thalidomide did not develop hematological toxicity, and 3 (30.0%) experienced gastrointestinal reactions. Among the 30 patients that received maintenance therapy, 12 (40.0%) experienced grades 1–2 AEs, and 6 (20.0%) experienced grades 3–4 AEs, which were caused by chidamide-related hematological toxicity.

### Survival of the patients in the PTCL subgroups

The 3-year OS and PFS of NKTCL, ALCL (ALK-), AITL, and PTCL-NOS decreased in the following order: 78.3% and 67.9%, 71.4% and 57.1%, 66.6% and 61.5%, and 52.0% and 27.2%, respectively. Among the four subgroups, NKTCL showed the highest efficacy, and the 3-year PFS was significantly higher than that of PTCL-NOS (*P*<0.05).

### Multivariate analysis for survival

Post-transplant maintenance treatment (PFS: hazard ratio [HR] 0.271, *P*<0.001; OS: HR 0.212, *P*=0.003) was associated with significantly prolonged PFS and OS. An IPI score >2 (PFS: HR 2.475, *P*=0.022; OS: HR 3.546, *P*=0.006) and PR status before transplantation (PFS: HR 4.413, *P*<0.001; OS: HR 6.352, *P*<0.001) were associated with significantly poor PFS and OS (**Table 3**).

**Table 3:** Multivariate analysis of the survival of 69 PTCL treated with ASCT.

Factors	3-year PFS			3-year OS		
	HR	95% CI	*P*	HR	95% CI	*P*
IPI score	2.475	1.137-5.386	**0.022**	3.546	1.442-8.721	**0.006**
Maintenance treatment	0.271	0.122-0.603	**<0.001**	0.212	0.076-0.597	**0.003**
Response before ASCT			**0.001**			**<0.001**
CR2 vs CR1/PR	1.358	0.295-6.255	0.695	0.866	0.106-7.053	0.893
PR vs CR1/CR2	4.413	2.015-9.667	**<0.001**	6.352	2.451-16.465	**<0.001**
PIT score	1.489	0.481-4.608	0.490	|	|	|
Gender	0.571	0.270-1.208	0.143	|	|	|
Extranodal involvement	|	|	|	2.216	0.471-10.411	0.314
B symptoms	|	|	|	1.607	0.689-3.744	0.272

PTCL, Peripheral T-cell lymphoma; ASCT, autologous hematopoietic stem cell transplantation; CR1, first complete remission; CR2, second complete remission; PR, partial remission OS, overall survival; PFS, progression-free survival; HR, Hazard risk; 95% CI, 95% confidence interval; B symptoms, fever, night sweats, weight loss; IPI score, International Prognostic Index; PIT, Prognostic Index for T-cell lymphoma. bold font, *P*<0.05.

## Discussion

In this retrospective study, we aimed to analyze the efficacy of ASCT and post-transplant maintenance therapy in PTCL. Excluding the PTCL patients who were not eligible for ASCT or were less fit for this study, 69 patients with PTCL who underwent ASCT were finally enrolled. The results suggested that patients with CR had favorable survival with ASCT. Patients with PR had worse outcomes than those with CR; however, post-transplant maintenance therapy improved the survival of patients with PR.

Patients with PTCL who only received chemotherapy had poor outcomes, with a 5-year OS of 20–35% (2). ASCT might be an effective regimen to improve survival, with a 3-year OS of 70% and a 3-year PFS of 39% (5), and produced better outcomes in patients with CR (6–8). In our study, the observed overall 3-year OS was 67.8% and the 3-year PFS was 53.0%. In addition, patients with CR1 had significantly better outcomes than those with PR (3-year OS: 85.3% vs. 38.4%, *P*<0.001; 3-year PFS: 65.4% vs. 32.0%, *P*=0.001). The 3-year OS and PFS of patients with CR2 were 80.0% and 60.0%, respectively, slightly lower than those of patients with CR1 and better than that of patients with PR. The results revealed that patients with PTCL who underwent ASCT, particularly those who achieved CR, had better outcomes than those who underwent chemotherapy alone. The 3-year OS was similar to other studies, but the 3-year PFS was higher in our cohort, which might be related to the fact that some patients underwent post-transplant maintenance therapy.

The high relapse rate is one of the reasons for poor outcomes in patients with PTCL, and patients with PR are more prone to relapse. Several studies have reported that the administration of HDAC to patients with relapsed or refractory PTCL improved survival (9–11). A relevant study demonstrated that the overall 2-year OS was only 35.0%. A total of 23.4% of patients achieved CR after chidamide administration, and these patients had better survival, with a 2-year OS of 69.4% (12). Another study reported favorable survival (2-year OS: 79.1%; 2-year PFS: 67.5%) with maintenance therapy after first-line chemotherapy for PTCL (4). To reduce the relapse rate after transplantation in our cohort, 56 patients who met one of the criteria of stages III–IV, IPI score > 2, and who did not achieve CR after transplantation were recommended for post-transplant maintenance therapy. However, patients with earlier transplantation had few maintenance therapy drugs to choose from, and some refused maintenance therapy. Only 30 patients (19 with CR1, 2 with CR2, and 9 with PR) eventually received maintenance treatment, while the remaining 39 did not. We observed that relapse and progression were more likely to occur within 2 years after transplantation, and the median time of relapse and progression between the groups with and without maintenance treatment was similar (8 months vs. 7 months). The relapse and progression rates were lower in the maintenance group than in the non-maintenance group, both within (26.7% vs. 38.5%) and after 2 years (3.3% vs. 7.7%). In addition, the post-transplant maintenance group in the entire cohort showed better OS (86.0% vs. 54.2%, *P*=0.004) and prolonged PFS (76.7% vs. 37.5%, *P*=0.004) than the non-maintenance group, similar to the findings of another study (13). This suggested that post-transplant maintenance treatment for 2 years was reasonable and effective.

Our study revealed that the disease remission status before transplantation significantly affected survival. This suggests that the effects of maintenance therapy might vary among patients with different disease remission statuses. Therefore, we further separately analyzed the role of maintenance therapy in patients with CR1, CR2, and PR. We observed that maintenance therapy slightly improved the survival of patients with CR1 and CR2. However, post-transplant maintenance therapy for patients with PR reduced the relapse rate (33.3% vs. 78.7%) and significantly improved survival (3-year OS: 66.7% vs. 21.9%, *P*=0.007; 3-year PFS: 66.7% vs. 12.5%, *P*=0.004). Consequently, patients with PR benefited more from post-transplant maintenance therapy.

All patients in this study tolerated the post-transplant maintenance therapy. Although 6 (20.0%) patients experienced grades 3–4 AEs, all of which were chidamide-related hematologic toxicities, they all recovered after supportive treatment and continued maintenance therapy without complications related to infection or bleeding. The remaining grades 1–2 AEs mainly included fatigue and gastrointestinal reactions.

This study had several limitations. First, this was a retrospective study with a long follow-up period. Further, 11 patients received ASCT 10 years before this study was conducted. These patients had few maintenance therapy drugs to choose from, and the treatments during transplantation and chemotherapy drugs were different, which could have created an unavoidable bias. Second, compared to prospective studies, retrospective studies are more likely to be influenced by patients’ intentions. Thirteen patients in this study refused maintenance therapy for financial reasons, and 3 for other reasons. Third, the sample size of this study was small, and only 5 patients had a CR2 status before transplantation. The diversity and number of pre-transplant disease statuses were insufficient, and the results were potentially biased. Therefore, a follow-up study with a larger sample size is required to reduce the effects of these confounding factors.

In conclusion, ASCT is an effective regimen for treating PTCL, especially in patients who achieve CR. Patients with PR hardly benefit directly from ASCT; however, post-transplant maintenance therapy effectively reduces relapse and improves the OS and PFS of these patients.

## Data availability statement

The raw data supporting the conclusions of this article will be made available by the authors, without undue reservation.

## Author contributions

JZ designed the study, assisted in data analysis, and edited the manuscript. ZH performed the data collection and analysis and drafted the manuscript. YS, YFL, KZ, QL, YF, YZh, YYL, and BF assisted in data analysis. ZL, JW, RG, YZu, HZ, ZY and FY assisted in data collection. All authors contributed to the article and approved the submitted version.

## References

[1] Wudhikarn K Bennani NN. How to sequence therapies in peripheral T cell lymphoma. Curr Treat Options Oncol (2021) 22(9):74. doi: 10.1007/s11864-021-00873-w 34213653

[2] Fossard G Broussais F Coelho I Bailly S Nicolas-Virelizier E Toussaint E. Role of up-front autologous stem-cell transplantation in peripheral T-cell lymphoma for patients in response after induction: an analysis of patients from lysa centers. Ann Oncol (2018) 29(3):715–23. doi: 10.1093/annonc/mdx787 29253087

[3] Ellin F Landström J Jerkeman M Relander T. Real-world data on prognostic factors and treatment in peripheral T-cell lymphomas: a study from the Swedish lymphoma registry. Blood (2014) 124(10):1570–7. doi: 10.1182/blood-2014-04-573089 25006130

[4] Guo W Wang X Li J Yin X Zhao Y Tang Y. Chidamide maintenance therapy following induction therapy in patients with peripheral T-cell lymphoma who are ineligible for autologous stem cell transplantation: case series from China. Front Oncol (2022) 12:875469. doi: 10.3389/fonc.2022.875469 35747802 PMC9209709

[5] Schmitz N Truemper L Bouabdallah K Ziepert M Leclerc M Cartron G. A randomized phase 3 trial of autologous vs allogeneic transplantation as part of first-line therapy in poor-risk peripheral T-nhl. Blood (2021) 137(19):2646–56. doi: 10.1182/blood.2020008825 PMC9635528 33512419

[6] d'Amore F Relander T Lauritzsen GF Jantunen E Hagberg H Anderson H. Up-front autologous stem-cell transplantation in peripheral T-cell lymphoma: nlg-T-01. J Clin Oncol (2012) 30(25):3093–9. doi: 10.1200/JCO.2011.40.2719 22851556

[7] Wu M Wang X Xie Y Liu W Zhang C Ping L. Outcome and prospective factor analysis of high-dose therapy combined with autologous peripheral blood stem cell transplantation in patients with peripheral T-cell lymphomas. Int J Med Sci (2018) 15(9):867–74. doi: 10.7150/ijms.23067 PMC6036090 30008598

[8] García-Sancho AM Bellei M López-Parra M Gritti G Cortés M Novelli S. Autologous stem-cell transplantation as consolidation of first-line chemotherapy in patients with peripheral T-cell lymphoma: a multicenter GELTAMO/FIL study. Haematologica (2022) 107(11):2675–84. doi: 10.3324/haematol.2021.279426 PMC9614542 35320921

[9] Shi Y Jia B Xu W Li W Liu T Liu P. Chidamide in relapsed or refractory peripheral T cell lymphoma: a multicenter real-world study in China. J Hematol Oncol (2017) 10(1):69. doi: 10.1186/s13045-017-0439-6 28298231 PMC5351273

[10] O'Connor OA Horwitz S Masszi T Van Hoof A Brown P Doorduijn J. Belinostat in patients with relapsed or refractory peripheral T-cell lymphoma: results of the pivotal phase II BELIEF (CLN-19) study. J Clin Oncol (2015) 33(23):2492–9. doi: 10.1200/JCO.2014.59.2782 PMC5087312 26101246

[11] Foss F Horwitz S Pro B Prince HM Sokol L Balser B. Romidepsin for the treatment of Relapsed/Refractory peripheral T cell lymphoma: prolonged stable disease provides clinical benefits for patients in the pivotal trial. J Hematol Oncol (2016) 9:22. doi: 10.1186/s13045-016-0243-8 26965915 PMC4785666

[12] Liu W Zhao D Liu T Niu T Song Y Xu W. A multi-center, real-world study of chidamide for patients with relapsed or refractory peripheral T-cell lymphomas in China. Front Oncol (2021) 11:750323. doi: 10.3389/fonc.2021.750323 34804937 PMC8602952

[13] Liu H Dong S Zeng Y Gao L Zhang X. Maintenance therapy with chidamide prevents from postoperative recurrence after autologous hematopoietic stem cell transplantation for peripheral T cell lymphoma. J Army Med Univ (2020) 42(17):1724–9. doi: 10.16016/j.1000-5404.202006020

