# Supplementary material for: A retrospective study of autologous hematopoietic stem cell transplantation for peripheral T-cell lymphoma: pre-transplant patients with partial remission benefit from post-transplant maintenance therapy
Source: Front Oncol. 2023 May 15;13:1162413. doi: 10.3389/fonc.2023.1162413 (PMC10225554; doi:10.3389/fonc.2023.1162413)
Supplement: Supplementary file 1 [file DataSheet_1.docx]

Excluded refractory patients (N=81)

Exclude cases with allogeneic hematopoietic stem cell transplantation (N=11)

Cases disagreeing transplantation (N=59)

PTCL patients with ASCT (N=69)

Excluded age> 65, ECOG> 2, organ dysfunction or activity infection cases (N=109)

Eligible for transplantation cases (N=139)

Total PTCL patients in our center from November 2001 to November 2021 (N=436）

The subtypes of more than 5 cases in PTCL (N=177)

Excluded the ALK+ALCL subtype and the subtypes of less than 5 cases in PTCL (N=107)
